# Emotional Intelligence and Problem Solving Strategy: Comparative Study Basedon "Tower of Hanoi" Test

**Published:** 2012

**Authors:** Zahra Arefnasab, Hosein Zare, Abdolreza Babamahmoodi

**Affiliations:** 1 Payam-Noor University, Tehran, Iran.

**Keywords:** Emotional Intelligence, Problem Solving Strategies, Tower of Hanoi Test

## Abstract

**Objective: **The aim of this study was to compare problem solving strategies between peoples with high and low emotional intelligence (EI).

** Methods**: This study is a cross sectional descriptive study.The sample groups include senior BS& BA between 20-30 years old into two with high and low emotional intelligence, each group had 30 subjects.Data was analyzed with non-parametric chi square test for main dependent variable (problem solving strategies) and accessory dependent variables(manner of starting and fulfillmentof the test).The Independent two group T-test was used for analyzing other accessory dependent variables(Number of errors and total time used for fulfillment of the test).

**Results**: There was a significant difference between two groups in “number of errors” (t=-3.67,p=0) and “total time used for fulfillment of the test”(-6.17,p=0) and there was significant relation between EI and “problem solving strategies” (χ2=25.71, p<0.01) and (Cramer's v = 0.65, p<0.01) .Also there was significant relation between EI and “fulfillment of test” (χ2=20.31, p<0.01) and (φ=0.58, p<0.01). But the relation between EI and "manner of starting the test" was not significant (χ2=1.11, p=0.29). Subjects with high EI used more “insightful” strategy and subjects with low EI used more “trial- error” strategy. The first group completed the test more rapidlyand with fewer errors, compared with the second group. In addition the first group was more successful in performing the test than the second one.

**Conclusion**: People with high EI significantly solve problems better than people with lowEI.

## Introduction

Cognition or mental activity applies to acquisition, storing and transformation of knowledge and includes of broad domain of mental processing like perception, memory, language, problem solving and other factors (1). Problem solving is a cognitive processing directed at transforming a given situation into a goal situation when no obvious method of solution is available to the problem solver (2).

The problem solving circle includes distinguishing the problem, definition of problem, strategy making for problem, organizing information about problem, allocation of resources, and inspection on problem solving and evaluation of problem solving (3). After primary problem solving theory of Thorndike and Gestalt approach, in 1972 Newell and Simon argued that it is possible to produce systematic computer simulations of human problem solving. They specified the problem solving strategy in a computer program. According to them the complexity of most problems means that we rely on heuristics. Heuristics can be contrasted with algorithms, to lead to problem solution. The most important of various heuristic methods is means-ends analysis (2).The next theory is “representation change” theory of Ohlsson. He attempted to incorporate key aspects of gestalt approach into an information processing. According to him “insight occurs in the context of impasse, which is unmerited in the sense that the thinker is, in fact, component to solve the problem”. The Gestaltists claimed that insight involves special processes, and so is very different from normal problem solving. Insight occurs suddenly and does not involve the gradual accumulation of information (4).Insight or ah-ha filling might activate the posterior cingulate cortex (5).

Cognitive psychology is still somewhat influenced by the computer analogy or metaphor.This approach does not lend itself readily to an examination of the relationship between cognition and emotion. However there has been a rapid increase in the number of cognitive psychologists working in the area of cognition and emotion. Major topics on this field are:

1- The effect of cognitive appraisal on emotional experience 

2- Emotional regulation

3- The effect of emotion on cognition

4- Cognitive biases associated with anxiety and depression (4).

One of the concepts related to emotion and emotional management is “emotional intelligence”. Among all the theories about “emotional intelligence”, those proposed by Mayer and Salovey, Bar-on and Goleman have generated the most interest in terms of research and application.Each of their theoretical paradigms conceptualizes “emotional intelligence” from one of following perspectives: as a form of pure intelligence consisting of cognitive ability only (6).Salovey and Mayer (1990, 1997) defined“emotional intelligence” as the ability to perceive, respond and manipulate emotional information without necessarily understanding it and the ability to understand and manage emotions without necessarily perceiving feelings well or fully experiencing them. It is divided into four branches. The first branch is emotional perception, which includes the ability to identify emotion in one’s physical states, feelings and thoughts; the ability to identify emotions in other people, designs, artwork, through language, sound, appearance, and behavior; the ability to express emotions accurately, and to express the needs related to those feelings; the ability to discriminate between accurate and inaccurate, or honest versus dishonest expressions of feelings.The second branch is emotional assimilation, which includes emotion-prioritized thinking by directing attention to important information. Emotional states differentially encourage specific problem-solving approaches such as when happiness facilitates inductive reasoning and creativity. The third branch is emotional understanding, which includes the ability to label emotions and recognize relations among the words and the emotions themselves, such as the relation between liking and loving; the ability to interpret the meanings that emotions convey regarding relationships.The fourth branch is emotion management, which includes the ability to stay open to feelings, both those that are pleasant and those that are unpleasant; the ability to reflectively engage or detach from an emotion depending upon it being judged to be informative or utility; the ability to reflectively monitor emotions in relation to oneself and others; the ability to manage emotion in oneself and others by moderating negative emotions and enhancing pleasant ones, without repressing or exaggerating information they may convey(6).

Many researchers have emphasized onthe role of emotion and emotional management in well being and success; ever, some believe that 2/3 of capacities for successfulness is emotional capacities(7) and 90 percent of successfulness in leadership is attributed to emotional intelligence capacity(8). In sum, emotional capacity is the best discriminatingfactor between ordinary and intelligent people (9).

According to above-mentioned studies, it can be concluded that recent studies have been focused on the effects of emotional management on cognitive processing.In addition we showed that other studies have indicated the role of emotion and emotional management in approach to a problem and problem solving.This study according to mentioned studies was designed to assess interaction between emotional management and problem solving strategies. 

Furthermore, we wanted to know if people with different emotional intelligence have differentapproach in their problem solving strategy.

## Materials and Methods

*Subjects:*

In this study 250 of senior BS& BA students of different universities in Tehran city, with the age range of 20-30 years old, participated voluntarily in this study. All subjects completed Bar-on questionnaire of emotional intelligence (EI-i). According to the result of the completed EI-i questionnaire, after test, the subjects were divided into two 30-member groups: high EI score group (with mean score +one standard deviation) and low EI score group (withmean score- one standard deviation) and subjects between two levels wereomitted. Then we compared Performance of Each group in Bar-On EI questionnaire and The Tower of Hanoi test.

*Bar-On EI questionnaire:*

EI-i is a self-report measure of emotionally and socially intelligent behavior that provides an estimate of emotional-social intelligence. This is the most widely used measure of emotional-social intelligence to date (10).The EI-i consists of 133 items in the form of short sentences and employs a 5-point response scale with a textual response format ranging from"very seldom or not trueof me" (1) to "very often true of me or true of me"(5) . The individual’s responses render a total EI score and scores on the following 5composite scales that comprise 15 subscale scores: Intrapersonal (comprising Self-Regard, Emotional Self-Awareness, Assertiveness, Independence, and Self-Actualization); Interpersonal (comprising Empathy, Social Responsibility, and Interpersonal Relationship); Stress Management (comprising Stress Tolerance and Impulse Control); Adaptability (comprising Reality-Testing, Flexibility, and Problem-Solving); and General Mood (comprising Optimism and Happiness) (11).

This questionnaire’s standard was evaluated and determined by Dehshiri and Samuie on Tehran and Esfahan universities, respectively. In Dehshiri’s study, test-retest reliability score was 0.735 and Cronbachۥs coefficient Alpha score was 0.733. These scores were determined in Samuie study as 0.88 and 0.93, respectively (12).

*The Tower of Hanoi test:*

The Tower of Hanoi test is one of the famous tests in the field of problem solving. This test has numerous versions. In this study we use the most common version with 3 bars and 4 disks. These 3 bars have the same size but 4 disks have different size from the smallest to the biggest. These disks were arranged with their size on the first bar. Subject must transfer these disks to the last bar in the same arrangement, in each transformation only one disk can be moved and never, the bigger disk can be placed on the smaller one (13).

In our study, the Tower of Hanoi test was done individually. First, the direction of the test was explained, and then each subject completed the test without time limitation. During completing the test, the subjects were under direct observation of the examiner. Some datawas recordedby the examiner, including: the manner of starting the test ; whether the subject began the test impulsively or with thinkingthe main variable, the manner of performing the test which consists of three strategy of problem solving; “trial- error”, “goal- mean analysis” and “insightful method”. In addition, the examiner recorded the number of errors (disobedience against rules), the total time of performing the test, and whether the test was finished with success or not.

*Statistics:*

This study is a cross sectional descriptive study. For analyzing the data nonparametric statistical test; chi square test and parametric independent two groups T-test were used. Emotional intelligence (High or low) was independent variable. Dependent variables include, “manner of starting the test” (impulsive or with thinking), “the problem solving strategies” (trial - error, mean-goal analysis and insightful method), “performing the test” (successfully or not successfully), “number of errors” and, “total time for fulfillment of the test”. 

**Table 1:** Descriptive statistics of EI questionnaire, number of errors and total time for completing Tower of Hanoi test

	**Low EI group(N=30)**	**High EI group(N=30)**
	Mean± SD	Mean± SD
**EI questionnaire**	246.43±4.69	3.39±367.50
**Number of errors**	2.23±0.14	0.17±1.4
**Total time**	11.85±0.49	7.030.60

## Results

Mean and standard deviation of EI questionnaire, “number of errors” and “the total spent time for completing the test” in “Tower of Hanoi” test can be seen in table 1.

Descriptive statistics of the other three dependent variables are presentedin table 2.

**Table 2:** Descriptive statistics of “Manner of starting the test”,” Problem solving strategy” and “Fulfillment of the test

**High EI group(n=30)**	**Low EI group(n=30)**	**Variables **	
**frequency(%)**	**frequency(%)**		
20(66.67)	16(53.33)	Impulsive	**Manner of starting the test**
10(33.33)	14(46.67)	With thinking	
12(40)	30(100)	Trial- error	**Problem solving strategy**
05(16.67)	00 (0)	Mean- goal	
13(43.33)	00(0)	Insightful	
29(96.67)	13(43.33)	Correctly	**Fulfillment of the test**
01(3.33)	17(56.67)	Not correctly	

Significance of the mentioned differences that was evaluated by independent two groups t- test (number of errors and total time for fulfillment test) are presented in table 3.

**Table3:** Results of two group independent t- test for two dependent variables; “number of errors” and “Total time for fulfillmentof the test

**Dependent variable**	**Mean differences**	**t**	**df**	**Sig**
Number of errors	0.83	3.76*	58	0.001
total time used for fulfillment of the test	4.82	6.27**	58	0.002

*, ** P<0.01

Also the relations between emotional intelligence and three variables mentioned in table 2 was evaluated by chi square test are presented in tables 4, 5 and 6. 

As can be seen from table 3, there are significant differences between the two groups regarding “number of errors” and “total time spent for completing the test”. Actually, high EI group had less “number of errors” ((t=3.76, p=0.001) and spent less time for completing the test (t=6.27, p=0.002), from statistically point of view.

**Table 4:** Summary of chi square test for relation between emotional intelligence and problem solving strategy

**Emotionalintelligence**	**Trial-error**	**Mean goal**	**Insightful**	**χ** ^2^	**p**	**Cramer's V**
**Low**	30	0	0	25.71	0.01	0.65*
**Low**	30	0	0			

*P<0.01

Results shown in the table 4 indicate that there is significant relation between emotional intelligence and “problem solving strategy” (χ2=25.71, p<0.01) and (Cramer's v = 0.65, p<0.01).

**Table 5:** Summary of chi square test for relation between emotional intelligence and success in fulfillment of the test

**Emotional intelligence**	**Correctly**	**Not correctly**	**χ** ^2^	**p**	**φ**
**High**	29	01	20.31	0.01	0.58*
**Low**	13	17			

*p<0.01

Results shown in the table 5 indicate that there is significant relation between emotional intelligence and “success in fulfillment of the test” (χ2=20.31, p<0.01) and (φ =0.58, p<0.01) 

Results shown in the table 6 indicate that there is not significant relation between emotional intelligence and “manner of starting the test” (χ2=1.11, p=0.29) 

**Table 6:** summary of chi square test for relation between emotional intelligence and Manner of starting the test

**Emotionalintelligence**	**Impulsive**	**With thinking**	**χ** ^2^	**p**
**High**	20	10	1.11	0.29
**Low**	16	14		

## Discussion

The goal of this study was to compare problem solving strategies among people with high and low emotional intelligence. As indicated above, there is significant difference in problem solving strategies between people with high and low emotional intelligence. According to the result of our study, a significant percentage of subjects with high emotional intelligenceuse “insightful strategy”, compared with low emotional intelligencesubject. On the other hand, subjects with low emotional intelligence use “trial- error” strategymuch more than those with high emotional intelligence. 

When experience failure, individuals with high emotional intelligence have the capability of using stress management skill and try to form another representation of problem that can help to acquisition . insight and solve the problem, in contrast to low emotional intelligence individuals using “trial-error strategy” without considering alternatives in order to achieve the solution. Therefore, the first group solves the problem in less time than the second group. Furthermore, based on our test results, subjects with low emotional intelligence have more disobedience of test rule referred to test anxiety which cannot be under control completely by these subjects). In conclusion, high emotional intelligence individuals are more successful in completing the test, as previously shown by previous studies (7-9).This result is parallel to results of the studies that mentioned above, about the role of the emotions and emotional management in successfulness but not in problem solving strategies (7-9).

Although, to the best of our knowledge, the relationship between emotional intelligence and problem solving strategies has not been addressed, directly in previous studies, the role of emotional process in problem solving was evaluated and emphasized in many studies. In a research report conducted on the basis of Bar-On model about emotional intelligence, Stys and Brown said that defect in emotional intelligence can mean lack of success and the existence of emotional problems. Problems in coping with one’s environment is thought, by Bar-On, to be especially common among those individuals lacking in the subscales of reality testing, problem solving, stress tolerance, and impulse control(14). Channon showed that everyday problem-solving involves both non-social executive processes, social and emotional processes, and draws upon social and practical knowledge (15). Herman and Scherer reported that although cognitive skills are important to effective problem solving, the nature of these problems may also require emotional skills as well. The researchers in this study concluded that emotional intelligence may not equally influence all activities; highlighting the need to investigate which steps of the problem-solving process it does indeed impact (16).This subject was also evaluated in clinical cases in many studies supporting the relationship between emotion and problem solving strategies. It was showed that positive and negative emotions elicit distinguishable problem-solving strategies: Participants with negative emotions are more focused on the seeking and use of information (17).Fossati, Ergaise & Allialaire observed that there is a considerable association between problem-solving deficits and the mean duration of the depressive episode. Besides, they concluded that problem-solving abilities might be predictive of poorer outcome in patients with unipolar affective disorders (18).

On the other hand, the role of emotional skills on cognitive processes related to problem solving, including decision making and leadership, has been shown in other studies (9,19,20).

In a study about the relation between personality disorders and problem solving, showed that cluster A diagnoses were infrequent and not amenable to analyses. Of the cluster B diagnoses, Borderline predominated and was associated with an impulsive/careless problem solving style, as were Histrionic and Narcissistic. Of cluster C diagnoses, Avoidant was associated with negativity and low impulsive/careless problem solving style, and Dependent with negativity. Thus, the social problem solving profiles of specific personality disorders in clusters B and C mostly showed the expected associations with personality characteristics (21).

Other researchers studied the role of emotional skills in cognitive processes related to problem solving like decision making and leadership. These researches showed that this role is important (9,19,20).

In our study we directly worked on problem solving strategies and its relation with emotional intelligence but in mentioned studies almost this aspect was neglected. We tried to make relation between problem solving and emotional intelligence, if other studies approve findings of our study; it is not far away to produce new methods for increasing emotional intelligence by focusing on problem solving strategies.

Our study findings strongly support close association between emotion and cognition. It can be concluded that emotional intelligence skills training might improve cognitive processes like problem solving strategies considerably and therefore future studies need to be done in this regard. 

## Authors’ Contributions

ZAconceived and designed the study, collected the data, interpreted them, performed the statistical analysis and revised the manuscript. HZ participated in designing the evaluation conceived and designed the study. AB re-evaluated the data, revised the manuscript and performed the statistical analysis and drafted the manuscript. All authors read and approved the final manuscript.

## Notes

**Declaration of interest:** None.
